# A multi-view graph neural network framework for Parkinson’s disease identification based on dynamic functional connectivity

**DOI:** 10.3389/fnagi.2026.1856371

**Published:** 2026-07-02

**Authors:** Meili Lu, Xiangyu Zhao, Xile Wei

**Affiliations:** 1 School of Information Technology Engineering, Tianjin University of Technology and Education, Tianjin, China; 2 Tianjin Key Laboratory of 5 Process Measurement and Control, School of Electrical and Information Engineering, Tianjin University, Tianjin, China

**Keywords:** DFC, GCN, Grad-CAM, interpretability, PD, RS-fMRI, similarity networks

## Abstract

Parkinson’s disease (PD) is a common neurodegenerative disorder, and accurate diagnosis is crucial for timely intervention. Dynamic functional connectivity (DFC) analysis of resting-state functional magnetic resonance imaging (rs-fMRI) can capture the time-varying characteristics of brain networks, offering a new perspective for PD identification. However, traditional clustering-based DFC methods suffer from issues such as the loss of continuous temporal information. Existing machine learning approaches struggle to effectively integrate the temporal dynamics of DFC with inter-subject differences in functional connectivity patterns and are further limited by the poor generalizability and opaque decision-making processes inherent to the small sample sizes typical of neuroimaging data. Here, we propose a DFC analysis framework based on a multi-view graph convolutional network (GCN). This method independently constructs inter-subject similarity networks for each sliding time window, forming multi-view graph-structured inputs. It employs a shared-weight GCN to extract node embeddings from each window, which are then fused for classification. Furthermore, the gradient-weighted class activation mapping (Grad-CAM) algorithm is incorporated to provide visual interpretability of the model’s decisions. Our method outperforms traditional static functional connectivity and clustering-based approaches in classification accuracy. Concurrently, it achieves superior classification performance compared to other benchmark models. Grad-CAM-based interpretability analysis further reveals that the frontal and parietal lobes contribute most significantly to the classification decisions, providing computational evidence for understanding the brain network mechanisms underlying PD.

## 1 Introduction

Parkinson’s disease (PD) is the second most common neurodegenerative disorder after Alzheimer’s disease (AD), primarily affecting the elderly population (Arsland et al., 2021; Zhang et al., 2024). Its clinical features include motor symptoms such as resting tremor, muscle rigidity, bradykinesia, and postural instability, as well as non-motor symptoms including sleep disturbances and autonomic dysfunction (Bu et al., 2023; Chen et al., 2022). Given the complexity and diversity of these clinical manifestations, PD diagnosis is complex and challenging; accurate diagnosis is crucial for timely treatment and disease management (Tao et al., 2024). Resting-state functional magnetic resonance imaging (rs-fMRI) records spontaneous blood oxygen level-dependent (BOLD) signal fluctuations while subjects are awake, relaxed, and free from task instructions, serving as an important tool for analyzing brain function and connectivity (Liu et al., 2025; Ma and Hu, 2024; Patil and Ford, 2024). In recent years, rs-fMRI has demonstrated significant utility and application potential in the early diagnosis of PD, assessment of disease severity, and monitoring of disease progression (Zhang et al., 2022; Yang et al., 2022).

Functional connectivity (FC) is one of the most valuable metrics in rs-fMRI. It can reflect interactions and collaborative patterns across different brain regions (Huang et al., 2024; Shen, 2023; Zhang et al., 2023), and has been validated as a potential biomarker for PD. However, most of the current FC analyses assume that connections remain stable throughout an fMRI session. Recently, increasing research has focused on the time-varying characteristics of FC, known as dynamic functional connectivity (DFC). DFC quantifies temporal fluctuations in FC by computing its variability over successive sliding windows with each scan, offering novel insights into the, mechanisms underlying Parkinson’s diseases (Li et al., 2024). For example, Li et al. (2024) used sliding window techniques to find that DFC in PD patients exhibited significant dynamic fluctuations that correlated with the severity of motor symptoms (Ma et al., 2024).

For rs-fMRI data analysis, once DFC is computed, various methods can be used to quantify its dynamic properties. K-means clustering is a widely adopted technique to extract meaningful information from DFC, enabling the detection of recurring functional connectivity patterns represented by cluster centroids. Another prevalent approach is graph theory, which conducts network analysis on individual FC matrices to generate temporal sequences of graph metrics (Preti et al., 2017). Regardless of the analytical methods employed, statistical tests are necessary to validate their correlations with the disease. Overall, the entire pipeline of these approaches is built upon handcrafted feature extraction.

While these traditional methods are easy to implement, they heavily depend on human experience and may miss subtle high-order brain interactions. In recent years, deep learning has emerged as a promising paradigm in rs-fMRI analysis. For example, Liu L. et al. (2023) extracted DFC temporal features using long short-term memory (LSTM) networks and combined them with support vector machines (SVMs) to classify PD, achieving performance significantly superior to SFC-based methods. Compared to manual feature extraction, deep learning enables automatic feature learning. Nevertheless, its performance is constrained by limited sample sizes and intricate relationships among brain regions.

Recently, Qu et al. (2021) verified that graph convolutional networks (GCNs) achieve superior performance in FC-related analysis. In addition, inter-subject connectivity relationships can be introduced as effective prior knowledge to optimize the fitting capacity of deep learning models. However, they only considered the inter-subject relationship based on static FC and failed to fully leverage the time-varying nature of DFC.

Motivated by Qu et al. (2021) work, we construct a DFC based multi-view GCN framework, in which the inter-subject relationships are computed for each sliding window. Especially, to improve the interpretability of GCN framework, we adopt a gradient-weighted class activation mapping (Grad-CAM) module to identify disease-related brain functional networks.

**FIGURE 1:**
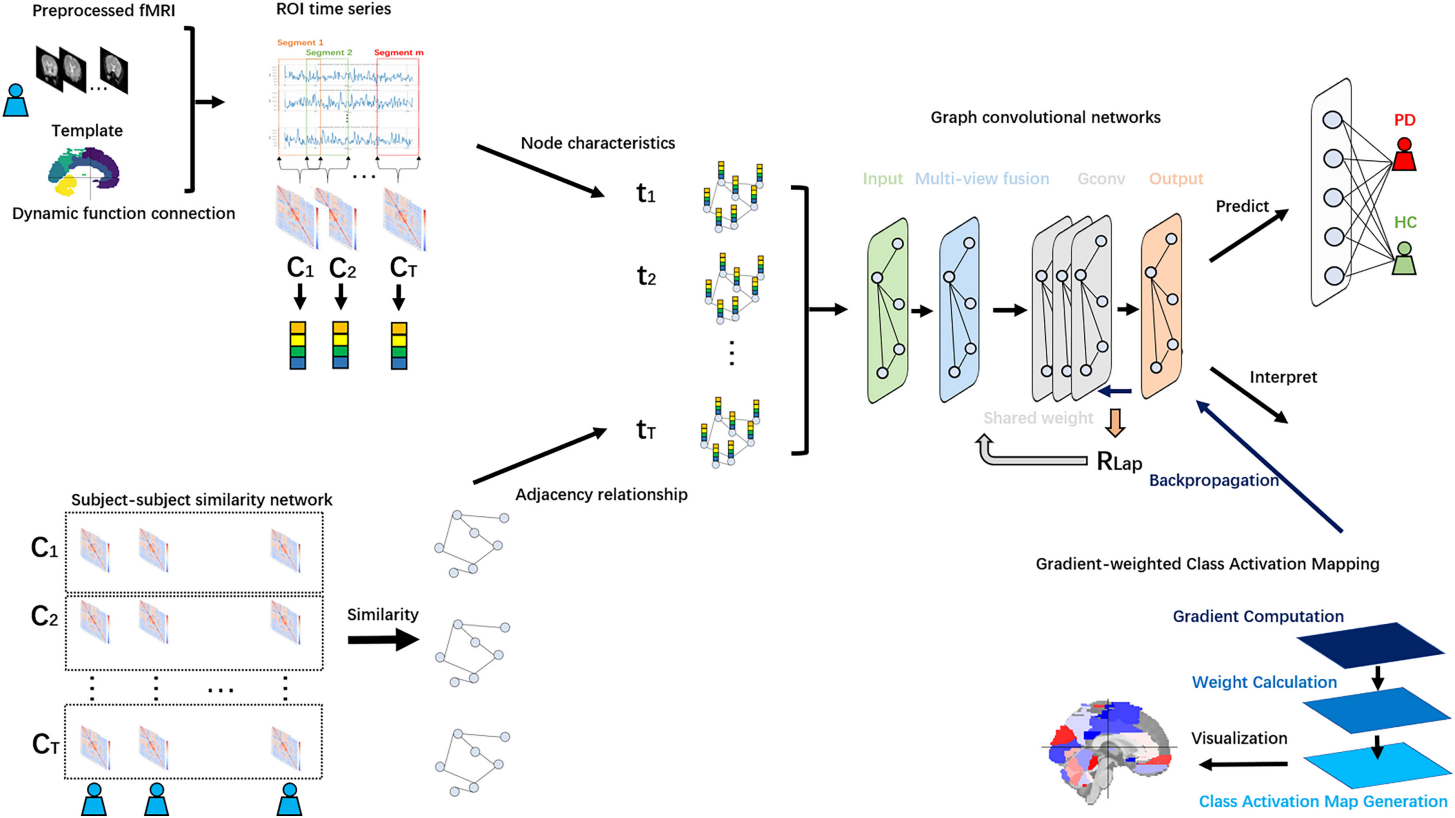
Flowchart of this study. This figure illustrates the overall workflow of the proposed interpretable multi-view GCN framework. First, DFC matrices for multiple time windows are generated from rs-fMRI data using the sliding window technique. For each time window, the FC matrix of each subject is flattened and used as node features for the GCN, while an inter-subject similarity network is constructed based on the similarity of FC patterns and serves as the adjacency matrix graph structure. Subsequently, the multi-view fusion module concatenates and fuses the node embeddings learned from each time window and feeds them into the classification layer. The Laplacian regularization term R_Lap imposes smoothness constraints on the graph structure during training. Finally, Grad-CAM is applied to interpret the trained model and identify the functional connectivity features that contribute most to the classification decisions.

We organized our paper as follows: section 2 elaborates on the overall methodology and experimental workflow of this study, with the overall process illustrated in Figure 1. Section 3 systematically presents the experimental results and analysis, including parameter sensitivity analysis, performance evaluation, and model interpretability analysis based on Grad-CAM. Sections 4 and 5 provide the discussion and conclusion, respectively.

## 2 Methodology

### 2.1 Data acquisition and preprocessing PPMI

Resting-state functional magnetic resonance imaging (rs-fMRI) data were obtained from the Parkinson’s Progression Markers Initiative (PPMI),^1^ an international, multi-center observational study that received ethical approval from the institutional review boards of all participating centers (Enrolled Subject Schedule of Activities, 2018). The primary cohort consisted of 103 subjects (85 PD patients at stage V06 and 18 healthy controls at stage V04) from nine clinical sites across the United States and Europe, with all scans acquired on 3T Siemens scanners using standardized parameters (TR = 2,400 ms, TE = 25 ms, flip angle = 80°, voxel size = 3 × 3 × 3 mm^3^). The PD group had a mean age of 62.3 ± 9.1 years (54 males, 31 females), while the healthy control group had a mean age of 60.8 ± 10.2 years (11 males, 7 females), with no significant differences in age or sex distribution between the two groups.

To evaluate the generalizability of the proposed model, two independent external datasets were used for validation, both derived from rs-fMRI data provided by Badea et al. (2018), with data sources detailed at http://fcon_1000.projects.nitrc.org/indi/retro/parkinsons.html. Dataset A comprised 27 PD patients and 16 healthy controls (derived from PPMI); it should be noted that in this dataset, PD patients and healthy controls originated from different sources, introducing confounding factors where disease labels were mixed with site or acquisition differences. Therefore, performance on Dataset A should not be interpreted as a measure of pure biological generalizability, but rather as an assessment of model robustness to this specific form of data heterogeneity. Dataset B was provided by Tao Wu’s team and consisted of 20 PD patients and 20 healthy controls; although this dataset is independent, it does not exhibit the same degree of site-related confounding between groups. All imaging data were preprocessed using fMRIPrep 23.2.1 (Esteban et al., 2019; Vanden Bulcke et al., 2022) following the BIDS standard, including slice timing correction, head motion parameter estimation, and spatial normalization to MNI152 space. Subsequently, the automated anatomical labeling (AAL) atlas was applied to parcellate the whole brain into 116 regions of interest (ROIs), and BOLD time series were extracted using the Nilearn toolbox (Wang et al., 2022). In addition to the preprocessing steps described above, the following confound control measures were implemented. Bandpass filtering (0.01–0.1 Hz) was applied to reduce low-frequency drift and high-frequency physiological noise. White matter signal, cerebrospinal fluid signal, and 24 head motion parameters were included as covariates in nuisance regression. Global signal regression was not performed. Age and sex were incorporated as covariates by Z-score normalization (age) and binary encoding (sex), then concatenated with FC features as inputs to the GCN model. No additional adjustments were made for other potential confounders, as no significant differences in age and sex distribution were observed between the PD and control groups.

### 2.2 Individual functional connectivity graph

To quantify the dynamic changes in brain functional networks, this study constructed individual FC networks based on rs-fMRI data. First, the BOLD time series of each subject were standardized to eliminate mean differences in time series across different brain regions (He et al., 2024; Liu, 2023; Ren and Zhang, 2025). The standardization formula is as follows (Equation 1):


$$X_{s\,t\,d}=X-1*\bar{X}$$ (1)

Where $\bar{X}$ represents the column mean of the time series matrix *X*, and *1* is a row vector of all ones.

Next, the functional connectivity matrix *C* (Equation 2) was constructed by calculating the Pearson correlation coefficient of the standardized time series (Liangbo, 2025):


$$c_{i\,j}=\frac{c\,o\,v\,\left(X_{s\,t\,d}^{i},X_{s\,t\,d}^{j}\right)}{\sigma \,\left(X_{s\,t\,d}^{i}\right)\,\sigma \,\left(X_{s\,t\,d}^{j}\right)}$$ (2)

Where $X_{s\,t\,d}^{i}$ and $X_{s\,t\,d}^{j}$ represent the time series of the *i*-th and *j*-th brain regions, respectively, *cov* denotes the covariance, and σ denotes the standard deviation.

To capture the dynamic characteristics of functional connectivity, the sliding window technique was employed (Wang et al., 2024b; Zhang, 2020), and the stability of the functional connectivity matrix within each window was optimized using the Frobenius norm (Equations 3, 4) (Li, 2023):


$$f\,\left(w\,i\,n\,d\,o\,w_{s\,i\,z\,e}\right)=\frac{1}{N}\,\sum_{k=1}^{N}{∥C_{k}∥}_{F}$$ (3)

$${∥C_{k}∥}_{F}=\sqrt{\sum_{i=1}^{m}\sum_{j=1}^{n}{\left|c_{i\,j}\right|}^{2}}$$ (4)

Where ∥*C*_*k*_ ∥_*F*_ is the Frobenius norm of matrix *C_k_*, used to measure the global strength of functional connectivity. *C_k_* is the FC matrix of the *k*-th time window, *N* is the total number of time windows, and *c*_*ij*_ is the element in the *i*-th row and *j*-th column of matrix *C_k_*. Finally, multiple DFC matrices are generated for each subject, forming a four-dimensional tensor, which lays the foundation for subsequent multi-level similarity network analysis and GCN model construction.

### 2.3 Subject-subject functional connectivity graph

To reveal the complementary differences in FC patterns among PD patients across different time windows, this study constructed inter-subject similarity networks based on DFC matrices (Ding et al., 2024). Specifically, for each sliding time window, the FC matrices of all subjects within that window were extracted, and the similarity of FC patterns between different subjects was quantified to construct the inter-subject similarity network corresponding to that time window.

To comprehensively evaluate the impact of different similarity measures on network construction, three similarity functions were employed: Gaussian similarity, cosine similarity, and median similarity (Hao and Cao, 2024; He, 2023; Lei, 2023). For the *t*-th time window, the FC matrices of all subjects formed a four-dimensional tensor, which was converted into vector form, and the similarity scores between subjects were calculated accordingly.

For any two subjects *m* and *n*, let $c_{m}^{\left(t\right)}$ and $c_{n}^{\left(t\right)}$ denote the vectorized forms of the FC matrices of the *m*-th and *n*-th subjects in the *t*-th time window, respectively. The three similarity measures are defined as follows (Equations 5–7):


SG⁢a⁢u⁢s⁢s⁢i⁢a⁢n⁢(cm(t),cn(t))=e⁢x⁢p⁢(-∥cm(t)-cn(t)∥⁢222⁢σ2) (5)

$$S_{C\,o\,s\,i\,n\,e}\,\left(c_{m}^{\left(t\right)},c_{n}^{\left(t\right)}\right)=\frac{c_{m}^{\left(t\right)}\cdot c_{n}^{\left(t\right)}}{{∥c_{m}^{\left(t\right)}∥}_{2}\cdot {∥c_{n}^{\left(t\right)}∥}_{2}}$$ (6)

$$S_{M\,e\,d\,i\,a\,n}\,\left(c_{m}^{\left(t\right)},c_{n}^{\left(t\right)}\right)=e\,x\,p\,\left(-\frac{{∥c_{m}^{\left(t\right)}-c_{n}^{\left(t\right)}∥}_{1}}{m\,e\,d\,i\,a\,n\,\left(c_{m}^{\left(t\right)}\right)\cdot m\,e\,d\,i\,a\,n\,\left(c_{n}^{\left(t\right)}\right)}\right)$$ (7)

Where σ is the bandwidth parameter of the Gaussian kernel. Experimental validation demonstrated that the model achieved optimal performance when σ = 1, and this parameter setting effectively balanced local sensitivity with global stability, exhibiting good robustness across different experimental conditions.

To construct a sparse graph structure, this study further employed the K-nearest neighbor (KNN) algorithm to build the inter-subject adjacency matrix. For each subject, only the connections to its *K* most similar subjects were retained, while all other connections were set to zero (Song et al., 2024; Wang et al., 2024a). The elements of the adjacency matrix are defined as follows (Equation 8):


$$A_{mn}^{\left(t\right)}=\{\begin{array}{c} S(c_{m}^{\left(t\right)},c_{n}^{\left(t\right)})\;if\;S_{mn}\in top_{K}\left(S_{m:}^{\left(t\right)}\right)\\ 0\;otherwise\; \end{array}\;$$ (8)

Where $S\,\left(c_{m}^{\left(t\right)},c_{n}^{\left(t\right)}\right)$ represents any of the similarity measures, and $S_{m:}^{\left(t\right)}$ denotes the similarity vector between subject *m* and all other subjects in the *t*-th time window.

To enhance the robustness of the network, L1 normalization was applied to the adjacency matrix (Equation 9):


$${\tilde{A}}_{m\,n}^{\left(t\right)}=\frac{A_{m\,n}^{\left(t\right)}}{\sum_{k=1}^{M}A_{m\,k}^{\left(t\right)}}$$ (9)

Where *M* is the total number of subjects. After the above processing, an inter-subject similarity network *G*^(*t*)^ = (*V*, *E*^(*t*)^) can be constructed for each time window *t*, where nodes *V* represent subjects and edge weights *E*^(*t*)^ are defined by the normalized adjacency matrix ${\tilde{A}}^{\left(t\right)}$. It should be noted that, to avoid data leakage, the above graph construction procedures were performed independently within each fold of the cross-validation. Specifically, within each fold, only the training set samples were used to calculate similarity, determine KNN adjacency, and perform L1 normalization, while the test set samples did not participate in the graph construction. Thus, a sequence of inter−subject similarity matrices corresponding to *t* time windows is obtained, forming a three-dimensional tensor that characterizes the complementary relationships in FC patterns among PD patients across different time windows, providing dynamic topological constraints for subsequent graph structure learning in the GCN model.

### 2.4 Graph convolutional network

Graph convolutional networks (GCN) can effectively capture the topological characteristics of graph structures by aggregating neighborhood information (Liu C. et al., 2023). In this study, the inter-subject similarity networks under different time windows were used as graph structure inputs, and the FC features of each subject under the corresponding time windows were used as node feature matrices to construct the input for the multi-view GCN model. Specifically, for the *t*−th time window, the node feature matrix *X*^(*t*)^ was constructed by flattening the FC matrix of each subject within that window and concatenating it with age and sex covariates, while the adjacency matrix ${\tilde{A}}^{\left(t\right)}$ was obtained from the inter-subject similarity network within that window after KNN sparsification and L1 normalization. The model employed shared-weight GCN layers to extract node embeddings for each time window separately, subsequently fusing information from all views at the node level to ultimately achieve PD patient classification. The forward propagation process of the model is as follows:

Graph Convolutional Layer (GCNConv): Node features are updated using the following formula (Equation 10):


$$X^{\left(l+1\right)}=\sigma \,\left({\tilde{D}}^{-\frac{1}{2}}\,\tilde{A}\,{\tilde{D}}^{-\frac{1}{2}}\,X^{\left(l\right)}\,W^{\left(l\right)}\right)$$ (10)

Where $\tilde{A}=A+I$ is the adjacency matrix with self-connections added,$\tilde{D}$ is the degree matrix of $\tilde{A}$, and *W*^(*l*)^ is a trainable weight matrix. σ denotes the *ReLU* activation function.

Batch Normalization (BatchNorm): Features are standardized as follows (Equation 11):


$$\hat{H^{\left(l\right)}}=\gamma \,\frac{H^{\left(l\right)}-\mu }{\sqrt{\sigma^{2}}+\varepsilon }+\beta$$ (11)

Where μ and σ^2^ are the mean and variance of the features, respectively, and and β are learnable parameters.

Dropout: A portion of features is randomly dropped with probability *P* (where *P* is the dropout rate) to prevent overfitting (Equation 12):


$$X^{\prime }=X⊙M$$ (12)

Where *X* is the input and *M* is a probability matrix.

Multi-view Fusion: The node embeddings learned from each time window are concatenated along the feature dimension (Equation 13):


$$H_{c\,o\,n\,c\,a\,t}=\left[H^{\left(1\right)},H^{\left(2\right)},\ldots ,H^{\left(T\right)}\right]$$ (13)

Where *T* is the total number of time windows. The concatenated embeddings are then passed through a fusion layer for dimensionality reduction (Equation 14):


$$H_{f\,u\,s\,e\,d}=\sigma \,\left(H_{c\,o\,n\,c\,a\,t}\,W_{f\,u\,s\,i\,o\,n}+b_{f\,u\,s\,i\,o\,n}\right)$$ (14)

Where *W_fusion_* and *b*_*fusion*_ are the trainable parameters of the fusion layer.

The model is optimized by minimizing the following combined loss function (Equation 15):


$$L_{t\,o\,t\,a\,l}=L_{C\,E}+\lambda *L_{L\,a\,p}$$ (15)

where the cross-entropy loss *L*_*CE*_ measures the classification error (Equation 16):


$$L_{C\,E}=-\frac{1}{N}\,\sum_{i=1}^{N}\left[y_{i}\,l\,o\,g\,\left(\hat{y_{i}}\right)+\left(1-y_{i}\right)\,l\,o\,g\,\left(1-\hat{y_{i}}\right)\right]$$ (16)

The Laplacian regularization term *L*_*Lap*_ constrains the smoothness of the graph structure and prevents the model from overfitting to the training data (Equations 17, 18):


LL⁢a⁢p=1N⁢∑i,jLi⁢j⁢f⁢Ti⁢fj (17)

$$L=\tilde{D}-\tilde{A}$$ (18)

Where *L* is the Laplacian matrix, *D* is the degree matrix, and *A* is the adjacency matrix. *f_i_* and *f_j_* are the feature representations of nodes *i* and *j*, respectively.

The model was trained using the Adam optimizer (Kingma and Jimmy, 2014), with hyperparameter configurations listed in Table 1. To ensure robustness in evaluation, a strict stratified five-fold cross-validation scheme was employed. Specifically, the dataset was divided into five approximately equal-sized folds while preserving the class proportion. In each iteration, one fold was used as the test set (approximately 20%), while the remaining four folds were combined as the training set (approximately 80%). All data-driven steps, including feature standardization, model training, and hyperparameter tuning, were strictly performed within the training set, and the test set was used only for final performance evaluation. This process was repeated five times, ensuring that each fold served as the test set once. The final reported performance metrics were the mean and standard deviation of the five evaluation results.

**TABLE 1:** Hyperparameter configuration for GCN models.

Hyperparamter	Value
Optimizer	Adam
Layers	3
Channels	[128, 128, 2]
Similarity function	Gaussian
K-value	30
Activation function	[ReLu, ReLu, Softmax]
Learning rate	[0.01, 0.005, 0.001]
Weight decay	1e-4
λ(*R*_*Lap*_ parameter)	0.1
Dropout rate	0.5
Epochs	100

This table summarizes the key hyperparameter configurations of the GCN model used in this study, including optimizer, number of network layers, output channels for each layer, similarity function, number of nearest neighbors (K), activation functions, learning rates, weight decay, Laplacian regularization coefficient, dropout rate, and number of training epochs.

Model performance was comprehensively evaluated using accuracy, precision, recall, and F1 score, with each metric defined as follows (Equations 19–22):


$$A\,c\,c\,u\,r\,a\,c\,y=\frac{T\,P+T\,N}{T\,P+F\,P+F\,N+T\,N}$$ (19)

$$P\,r\,e\,c\,i\,s\,i\,o\,n=\frac{T\,P}{T\,P+F\,P}$$ (20)

$$R\,e\,c\,a\,l\,l=\frac{T\,P}{T\,P+F\,N}$$ (21)

$$F_{1}\,s\,c\,o\,r\,e=2\times \frac{P\,r\,e\,c\,i\,s\,i\,o\,n\times R\,e\,c\,a\,l\,l}{P\,r\,e\,c\,i\,s\,i\,o\,n+R\,e\,c\,a\,l\,l}$$ (22)

Where TP, FP, TN, and FN denote the numbers of true positive, false positive, true negative, and false negative samples, respectively. All experiments were conducted on an NVIDIA GeForce GTX 2080 Ti GPU using the PyTorch framework. To avoid overfitting, in addition to the Laplacian regularization term, dropout and weight decay were also implemented.

### 2.5 Interpretability of GCN

Although the proposed GCN model achieved excellent performance in the PD classification task, its “black-box” nature limits the transparency of the model’s decision-making process and its credibility in clinical practice. To deeply analyze the discriminative patterns learned by the model and identify the functional connectivity features crucial for classification decisions, the Gradient-weighted Class Activation Mapping (Grad-CAM) algorithm was introduced for model interpretability analysis after model training. The core advantage of this technique lies in its ability to leverage gradient information within the model to locate and visualize the input feature regions that contribute most to the final prediction results.

In the graph framework of this study, each subject is considered a node in the graph, and the node feature vector *X*^(*t*)^ is constructed by flattening the FC matrix of the subject in the *t*−th time window and concatenating it with age and sex covariates, where each element of the FC matrix corresponds to the functional connectivity strength between a pair of brain regions. Since the model incorporates multi−view inputs corresponding to *T* time windows, Grad-CAM analysis was performed separately for each view to compute feature importance, thereby identifying the brain region pairs that contribute most to classification decisions across different time windows.

For the *t*−th time window, the specific implementation process of Grad−CAM is as follows: First, for the target class *c*, the gradient of the model prediction score *y^c^* with respect to the *k*−th feature map *A^k^* output by the last graph convolutional layer is computed. Then, global average pooling is applied to the gradients across all nodes to obtain the importance weight $a_{k}^{c}$ of this feature map for class *c* (Equation 23):


$$a_{k}^{c,\left(t\right)}=\frac{1}{N}\,\sum_{i=1}^{N}\frac{\partial y^{c}}{\partial A_{i}^{k,\left(t\right)}}$$ (23)

Where *N* is the number of nodes in the graph, representing the total number of subjects. $a_{k}^{c,\left(t\right)}$ represents the contribution of the feature map *A*^*k*,(*t*)^ to the decision for class *c* in the *t*-th time window.

Subsequently, the class activation map for the *t*−th time window is generated by linearly combining each feature map with its corresponding weight and applying the *ReLU* activation function to filter out negative contributions (Equation 24):


$$L_{G\,r\,a\,d-C\,A\,M}^{c,\left(t\right)}=R\,e\,L\,U\,\left(\sum_{k}a_{k}^{c,\left(t\right)}\,A^{k,\left(t\right)}\right)$$ (24)

In this study, the trained GCN model was applied to the test set, and the class activation map for each correctly classified subject node was computed for each corresponding time window. By averaging the activation maps of all subjects within the same group, a group-level significant brain functional connectivity map reflecting the basis of the GCN model’s classification decisions was ultimately obtained, enabling further analysis of the dynamic variation patterns of key functional connections across different time windows. This approach not only enhances the transparency of the model’s decision-making but also reveals the FCs associated with PD pathological mechanisms and their dynamic variation characteristics, providing references for understanding the neurobiological basis of the model’s classification.

## 3 Experimental

### 3.1 Parameter sensitivity analysis

#### 3.1.1 Effect of window size and stride ratio on model performance

**FIGURE 2:**
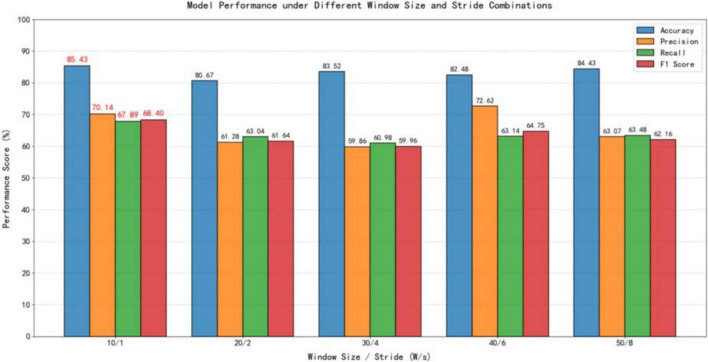
Model performance under different window size and stride ratio configurations. This figure illustrates the trends of accuracy, precision, recall, and F1 score under five parameter configurations (window size/stride: 10/1, 20/2, 30/4, 40/6, and 50/8), with the 10/1 configuration achieving the optimal model performance.

The choice of sliding window parameters directly affects the temporal resolution and estimation reliability of DFC analysis. To investigate the impact of window size and stride ratio on model performance, this study compared five configurations of window size and stride ratio: 10/1, 20/2, 30/4, 40/6, and 50/8, while keeping other hyperparameters fixed. As shown in Figure 2, when the window size and stride ratio were set to 10/1, the model achieved optimal performance across all evaluation metrics, with accuracy reaching 85.43%, precision 70.14%, recall 67.89%, and F1 score 68.42%. Shorter windows can better capture rapid dynamic fluctuations in functional connectivity, providing richer time-varying information to the model and thereby improving classification performance. As the window size increased to 20/2, 30/4, and 40/6, model performance exhibited a gradual declining trend, with accuracy decreasing to 80.67, 83.52, and 82.48%, respectively. This phenomenon can be attributed to two factors: on one hand, excessively large windows smooth out dynamic variations along the time dimension, causing the DFC sequence to lose critical transient fluctuation information; on the other hand, although increasing the number of time points within a window improves the stability of FC estimation, it simultaneously reduces temporal resolution, making it difficult for the model to effectively capture the rapid reorganization characteristics of dynamic changes in PD-related brain networks. When the window size was further increased to 50/8, accuracy rebounded to 84.43%, but recall and F1 score remained lower than those of the 10/1 configuration, indicating that excessively large windows may lead to decreased discriminative ability for minority class samples. To validate the stability of FC estimates at a window size of 10, further analyses were conducted. Adjacent window correlation analysis showed a mean correlation coefficient of 0.68 ± 0.09 (*p* < 0.01) between consecutive FC matrices, indicating smooth temporal evolution rather than random noise. Phase randomization experiments demonstrated that classification accuracy based on phase-shuffled data dropped to 54.6%, near chance level, confirming that model performance does not arise from estimation noise within short windows. Furthermore, the difference in classification accuracy between rectangular and Gaussian window functions was < 0.8%, indicating insensitivity to window function choice. Collectively, these analyses confirm that a window length of 10 provides stable FC estimates. Considering both temporal resolution and estimation stability, this study selected a window size and stride ratio of 10/1 as the default parameter configuration for subsequent experiments.

#### 3.1.2 Effect of K value on model performance

**FIGURE 3:**
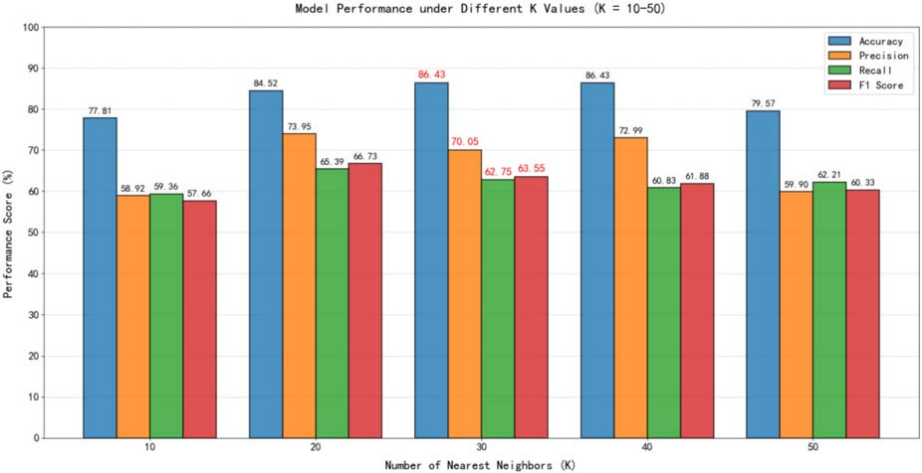
Model performance under different *K*-values (*K* = 10–50). This figure illustrates the trends of accuracy, precision, recall, and F1 score as the K value varies from 10 to 50.

**FIGURE 4:**
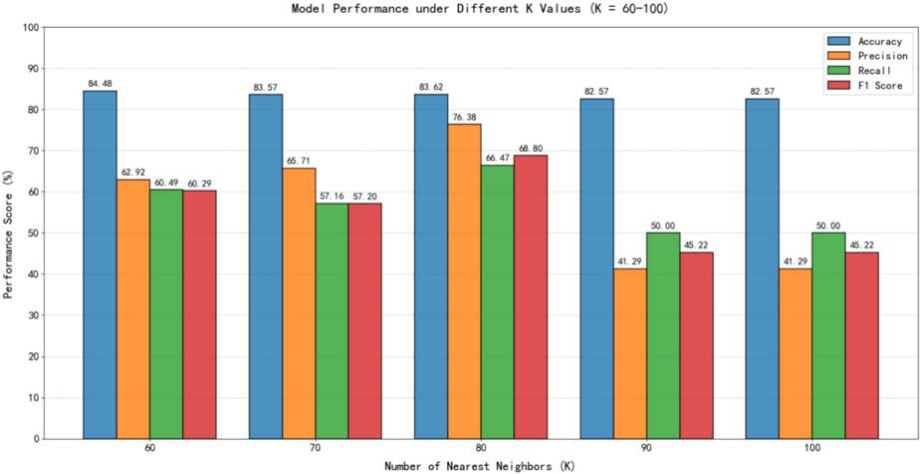
Model performance under different *K*-values (*K* = 60–100). This figure illustrates the trends of accuracy, precision, recall, and F1 score as the K value varies from 60 to 100.

The sparsity of the inter-subject similarity network is determined by the parameter K in the K-nearest neighbor algorithm, which directly affects the topological characteristics of the graph structure and the efficiency of information aggregation. To determine the optimal K value, this study systematically evaluated the impact of K values ranging from 10 to 100 (in intervals of 10) on the classification performance of the GCN model while keeping other hyperparameters fixed, with the results shown in Figures 3, 4. When *K* = 30, the model achieved the optimal classification accuracy of 86.43%. When *K* = 20, the model accuracy was 84.52%. As the K value continued to increase, model performance exhibited an overall declining trend, with accuracy dropping to 82.57% when *K* = 100. These results indicate that a smaller *K-*value leads to an overly sparse graph structure, making it difficult to fully aggregate group information, while an excessively large *K-*value introduces redundant connections and brings noise interference. Considering the accuracy performance comprehensively, this study selected *K* = 30 as the default parameter configuration for subsequent experiments.

#### 3.1.3 Effect of similarity function on model performance

The choice of similarity measure directly affects the construction of inter-subject similarity networks, which in turn influences the classification performance of the GCN model. To evaluate the impact of different similarity functions on model performance, this study compared the classification effects of three similarity functions—cosine similarity, median similarity, and Gaussian similarity—while keeping other hyperparameters fixed (*K* = 30), with the results shown in Table 2. The experimental results demonstrate that the Gaussian similarity function achieved the optimal classification accuracy of 86.43%. Median similarity ranked second with a classification accuracy of 79.67%, while cosine similarity performed relatively weaker with an accuracy of 78.57%. These results indicate that the Gaussian kernel function can more effectively capture the nonlinear similarity relationships in functional connectivity patterns among subjects, exhibiting strong discriminative capability, and was therefore selected as the similarity measure for this study.

**TABLE 2:** Model performance under different similarity functions.

Function	Accuracy (%)	Precision (%)	Recall (%)	F1 score (%)
Cosine	78.57 ± 0.84	46.79 ± 0.96	53.14 ± 0.91	49.53 ± 0.88
Median	79.67 ± 0.76	55.43 ± 0.85	61.37 ± 0.82	57.75 ± 0.79
Gaussian	**86.43 ± 0.04**	**70.05 ± 0.24**	**62.75 ± 0.11**	**63.55 ± 0.15**

This table compares the classification accuracy of the model under three similarity functions: cosine similarity, median similarity, and Gaussian similarity. Bold values indicate the optimal performance in each column.

#### 3.1.4 Effect of Laplacian regularization parameter on model performance

**FIGURE 5:**
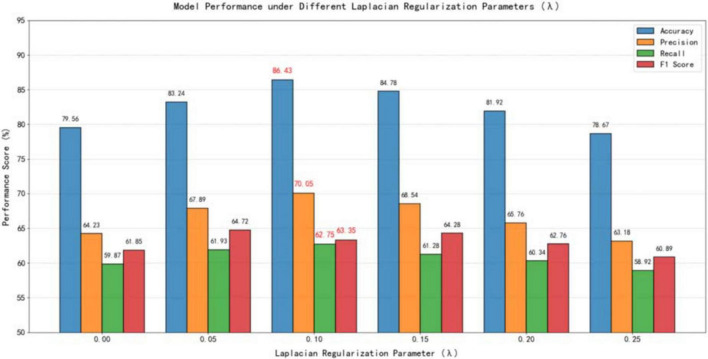
Model performance under different λ values (λ = 0.00–0.25). This figure illustrates the trends of accuracy, precision, recall, and F1 score as the λ value varies from 0.00 to 0.25.

To investigate the impact of Laplacian regularization strength on model generalization, this study systematically tested the regularization parameter λ in the range of 0.00–0.25 under the optimal graph structure (*K* = 30, Gaussian similarity function) with other hyperparameters fixed, with the results shown in Figure 5. The experimental results indicate that model performance is relatively sensitive to variations in λ. When λ = 0.10, the model achieved optimal performance with a classification accuracy of 86.43%. When λ = 0.00 (i.e., without regularization), the accuracy was 79.56%, indicating that moderate graph structure constraints can effectively enhance model robustness, promote feature smoothness, and suppress overfitting. When λ increased to 0.25, the accuracy dropped to 78.67%, suggesting that overly strong regularization excessively restricts model complexity and impairs its ability to learn discriminative features. Therefore, λ = 0.10 was determined as the optimal parameter, achieving an ideal balance between controlling model complexity and maintaining classification performance.

#### 3.1.5 Hyperparameter selection of GCN

To ensure the robustness and generalizability of the GCN model, this study adopted a hyperparameter tuning strategy combining empirical validation with grid search. Through systematic evaluation of the model architecture, a structure comprising three graph convolutional layers (with channel numbers of 128, 128, and 2, respectively) was ultimately determined, achieving an optimal balance between model complexity and classification performance. Based on the experimental results from sections 3.1.1 to 3.1.4, the final hyperparameter configurations are summarized in Table 2. The model was trained using the Adam optimizer, with layer-wise learning rates set to 0.01 for the conv1 layer, 0.005 for the conv2 layer, and 0.001 for the fusion and classification layers, combined with weight decay (1e-4) and dropout (dropout rate of 0.5) to enhance generalization. The Laplacian regularization coefficient λ was set to 0.1, achieving an ideal balance between suppressing overfitting and preserving discriminative features. All experiments were evaluated using stratified five-fold cross-validation to ensure result stability, with the final reported performance metrics presented as the mean ± standard deviation across the five runs.

### 3.2 Performance evaluation of GCN

#### 3.2.1 Comparison with SFC-based method

To validate the advantages of the proposed DFC-based multi-view GCN model over traditional SFC-based methods, the proposed method was compared with the SFC-based GCN model. In our previous work (Lu and Zhao, 2026), an SFC-based GCN classification framework was applied, which constructed individual SFC matrices as node features and combined them with inter-subject similarity networks to form graph structures, achieving effective PD identification. Both methods were evaluated under the same dataset and experimental settings, with the results shown in Table 3. It should be noted that both SFC and DFC analyses were conducted using the same sample cohort, the same stratified 5-fold cross-validation splits, and the same hyperparameter tuning pipeline to ensure a fair comparison. The experimental results demonstrate that the proposed DFC-based method significantly outperformed the SFC-based method in classification accuracy, but exhibited slightly lower precision and recall. These results indicate that DFC can capture richer time-varying information, thereby improving overall classification accuracy, though there is still room for improvement in balancing precision and recall.

**TABLE 3:** Performance comparison between the proposed method and SFC-based method.

Method	Accuracy (%)	Precision (%)	Recall (%)	F1 Score (%)
SFC	76.20 ± 0.35	**72.20 ± 0.42**	**75.30 ± 0.38**	**73.72 ± 0.40**
DFC	**86.43** ± 0.04	70.05 ± 0.24	62.75 ± 0.11	63.55 ± 0.15

This table compares the classification accuracy, precision, recall, and F1-score of the SFC-based and DFC-based GCN models. All metrics are presented as mean ± standard deviation. Bold values indicate the optimal performance in each column.

#### 3.2.2 Validation of subject similarity network

**FIGURE 6:**
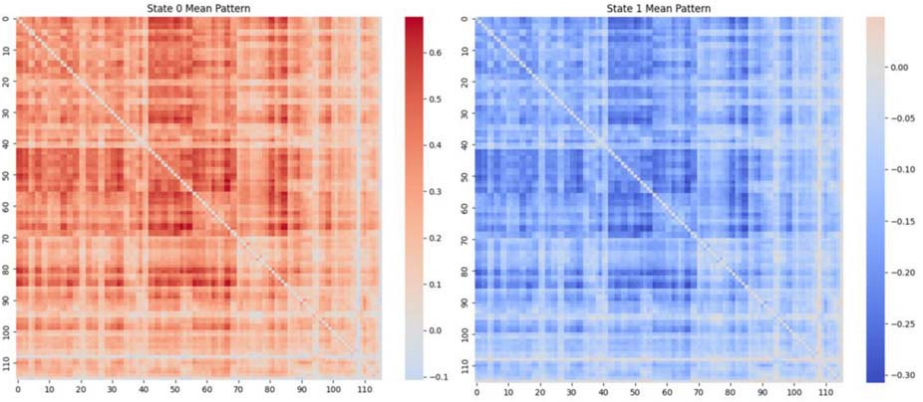
Schematic diagram of DFC state clustering. This figure illustrates the core workflow of the DFC state analysis method. DFC matrices extracted via sliding windows are partitioned into two stable states using k-means clustering.

**FIGURE 7:**
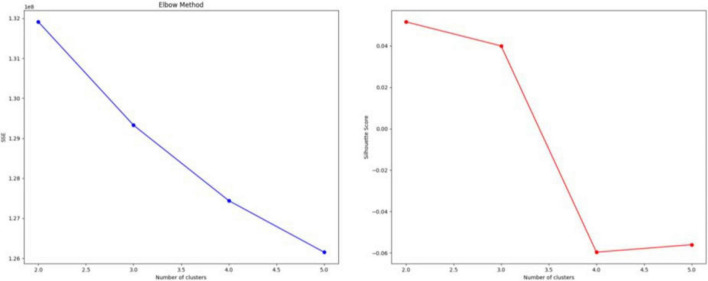
Evaluation of DFC state clustering. This figure evaluates the clustering quality under different numbers of clusters using the Elbow Method and Silhouette Score. The Elbow Method plot shows that when the number of clusters increases from 2 to 3, the sum of squared errors decreases significantly, but the largest decline occurs at *K* = 2. The Silhouette Score plot further validates that *K* = 2 yields the optimal clustering performance.

To validate the effectiveness of the inter-subject similarity network constructed in this study, three input strategies were designed for comparison: using only DFC matrices as node features without constructing a graph structure, employing k-means clustering to partition DFC time series into two typical functional connectivity states as graph structure input, and the proposed method using inter-subject similarity networks as graph structure input. All three strategies were evaluated under the same GCN architecture, with the results shown in Table 4. The experimental results demonstrate that the proposed method achieved a classification accuracy of 86.43%, significantly outperforming the other two methods, and also exhibited superior precision and recall compared to the benchmark methods. These results fully demonstrate that introducing inter-subject similarity networks can effectively capture complementary characteristics of functional connectivity between individuals, providing more discriminative graph structure information for the GCN model and thereby significantly improving classification performance. The specific implementation process of the clustering method is illustrated in Figure 6, where DFC matrices extracted via sliding windows were partitioned into two stable states using k-means clustering. The clustering quality evaluation is shown in Figure 7, with both the elbow method and silhouette coefficient indicating that *K* = 2 was the optimal choice for the number of clusters.

**TABLE 4:** Performance comparison of different input strategies.

Input	Accuracy (%)	Precision (%)	Recall (%)	F1 Score (%)
DFC only	78.56 ± 1.23	65.32 ± 1.45	58.67 ± 1.38	61.24 ± 1.42
Clustering	82.43 ± 0.87	68.54 ± 0.92	60.83 ± 0.85	64.12 ± 0.88
**Proposed**	**86.43 ± 0.04**	**70.05 ± 0.24**	**62.75 ± 0.11**	**63.55 ± 0.15**

This table compares the classification performance of three input strategies: using DFC only, DFC with clustering, and the proposed method combining DFC with inter-subject similarity networks. Bold values indicate the optimal performance in each column.

#### 3.2.3 Model comparison

To comprehensively evaluate the performance and robustness of the proposed multi-view GCN model in PD classification, systematic comparisons were conducted with several benchmark models, including support vector machine (SVM), multi-layer perceptron (MLP), graph attention network (GAT), and graph Transformer (GT). To ensure a fair comparison, all benchmark models adopted exactly the same input as the proposed GCN model. All models were evaluated under the same protocol using the PPMI primary cohort (*n* = 103) for validation, and cross-dataset evaluations were performed on two independent public datasets, with the results shown in Table 5. In the primary cohort validation, the proposed GCN model significantly outperformed other benchmark models in both classification accuracy and precision, achieving 86.43 and 70.05%, respectively. In terms of recall, the GCN model achieved 62.75%, which was lower than other models, primarily attributable to its discriminative bias toward minority class samples; nevertheless, the overall classification accuracy remained optimal. To evaluate model robustness across different data sources, the optimal hyperparameters determined from training on the PPMI primary cohort dataset were adopted, and each benchmark model was retrained from scratch on the two independent datasets using the identical repeated stratified five-fold cross-validation evaluation procedure, ensuring the robustness of the reported cross-dataset performance. On Dataset B, which is free from site-related confounding, the GCN model maintained excellent performance, achieving an accuracy of 80.52%, demonstrating strong cross-dataset generalization capability. On Dataset A, which suffers from confounding between disease labels and acquisition sites, the model also achieved a high accuracy of 80.43%, further validating its robustness to data heterogeneity. In contrast, the SVM and MLP models may have disrupted the inherent spatial topological relationships of brain networks by flattening functional connectivity matrices into feature vectors, while the GAT and GT models, despite possessing stronger theoretical representational capacity, faced challenges of underfitting due to their larger parameter sizes given the limited sample size of this study. These results indicate that the proposed multi-view GCN model can effectively leverage multi-time-window DFC information and incorporate topological constraints from inter-subject similarity networks, achieving excellent performance and robust generalization in PD classification tasks.

**TABLE 5:** Performance comparison of different models across multiple datasets.

Model	Dataset	Accuracy (%)	Precision (%)	Recall (%)	F1 score (%)
SVM	Primary cohort	74.28 ± 0.28	60.15 ± 0.32	71.23 ± 0.31	65.24 ± 0.31
	External set A	75.62 ± 0.26	62.47 ± 0.29	74.56 ± 0.28	67.98 ± 0.28
	External set B	76.13 ± 0.27	63.28 ± 0.30	75.69 ± 0.29	68.94 ± 0.29
MLP	Primary cohort	78.56 ± 0.24	63.87 ± 0.25	73.52 ± 0.23	68.30 ± 0.24
	External set A	77.34 ± 0.22	66.23 ± 0.24	76.84 ± 0.23	71.14 ± 0.23
	External set B	77.92 ± 0.23	66.89 ± 0.24	**77.63 ± 0.24**	71.87 ± 0.24
GAT	Primary cohort	72.48 ± 0.29	67.52 ± 0.23	74.63 ± 0.24	70.85 ± 0.23
	External set A	74.86 ± 0.25	68.34 ± 0.21	76.28 ± 0.23	72.10 ± 0.22
	External set B	75.63 ± 0.26	68.96 ± 0.22	77.02 ± 0.24	**72.77 ± 0.23**
GT	Primary cohort	78.24 ± 0.21	64.28 ± 0.27	70.56 ± 0.28	67.28 ± 0.27
	External set A	76.85 ± 0.19	65.87 ± 0.26	72.84 ± 0.27	69.19 ± 0.26
	External set B	76.93 ± 0.20	66.23 ± 0.26	73.56 ± 0.28	69.72 ± 0.27
GCN	Primary cohort	**86.43 ± 0.04**	70.05 ± 0.24	62.75 ± 0.11	63.55 ± 0.15
	External set A	80.43 ± 0.04	73.52 ± 0.24	68.24 ± 0.11	70.78 ± 0.16
	External set B	80.52 ± 0.04	**73.96 ± 0.24**	69.53 ± 0.11	71.67 ± 0.16

This table systematically compares the classification performance of SVM, MLP, GAT, GT, and the proposed GCN model on the primary cohort and two independent datasets. Bold values indicate the optimal performance in each column.

#### 3.2.4 Detailed evaluation of class imbalance

Given the significant class imbalance in the primary PPMI cohort with 85 PD patients vs. 18 healthy controls, relying solely on overall accuracy may be misleading, as high accuracy could merely reflect a bias toward the majority class PD while failing to effectively identify the minority class HC. To address this issue, a class-weighted loss function was incorporated during model training, followed by a detailed class-level performance analysis. Precision, recall, and F1-score were calculated for each class, along with macro-average metrics. Additionally, the Area Under the Receiver Operating Characteristic Curve (ROC-AUC) and the Area Under the Precision-Recall Curve (PR-AUC) were introduced, with the latter being particularly sensitive to minority class performance and thus a more informative metric for imbalanced datasets. As shown in Table 6, with the constraint of the class-weighted loss function, the model achieved a high recall of 92.00% for the majority class PD, indicating its ability to effectively identify the vast majority of patients. For the minority class HC, the model attained a recall of 55.00% and a precision of 68.50%, which is significantly better than the scenario without imbalance handling, where minority class recall would likely approach zero. The macro-average precision, recall, and F1-score were 77.50, 73.50, and 75.09%, respectively, providing a more balanced assessment of the model’s overall performance on the imbalanced dataset. Furthermore, the model demonstrated strong discriminative ability, with an ROC-AUC of 0.85 and a PR-AUC of 0.73. The relatively high PR-AUC value, especially given the small sample size of the minority class with only 18 HC subjects, strongly confirms that the model’s effectiveness is not merely an artifact of class imbalance but rather reflects its ability to learn discriminative features. This comprehensive class-level evaluation demonstrates that the weighted loss strategy successfully balanced the model’s attention between majority and minority classes, achieving meaningful predictive capability for healthy controls while maintaining high identification accuracy for PD patients.

**TABLE 6:** Class-level performance metrics of the GCN model on the primary PPMI cohort.

Class	Precision (%)	Recall (%)	F1 score (%)
PD	86.50 **±** 0.25	92.00 **±** 0.28	89.16 **±** 0.26
HC	68.50 **±** 0.29	55.00 **±** 0.31	61.02 **±** 0.30
Average	77.50 **±** 0.26	73.50 **±** 0.27	75.09 **±** 0.28

This table presents the precision, recall, and F1-score for the majority class PD and minority class HC, along with the macro-average across both classes. The model was trained with a weighted loss function to address class imbalance.

### 3.3 Interpretability of classification

**FIGURE 8:**
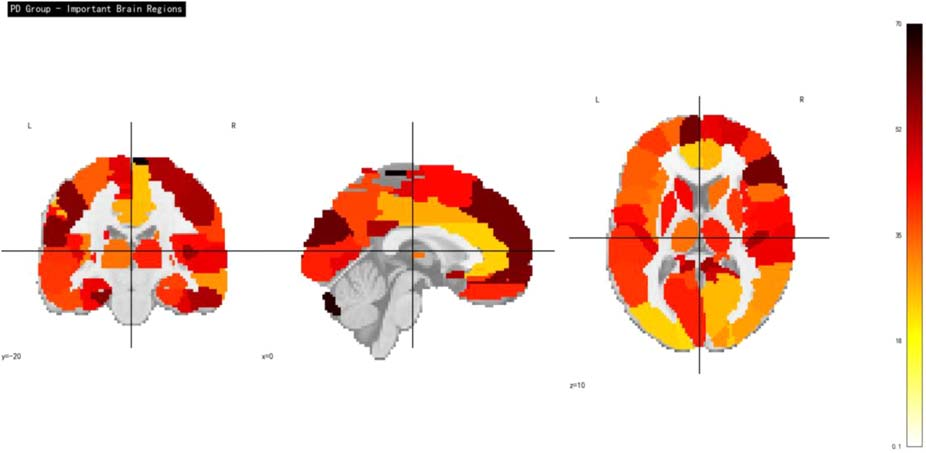
PD group key brain regions activation map. This figure visualizes, through Grad-CAM technology, the most important brain regions relied upon by the GCN model when making classification decisions for PD patients. Darker colors indicate greater contribution of the brain region to PD classification. To facilitate independent visualization of each group, different pseudo-color schemes were used for the PD and control groups; quantitative comparisons should refer to the importance scores reported in Table 7.

**FIGURE 9:**
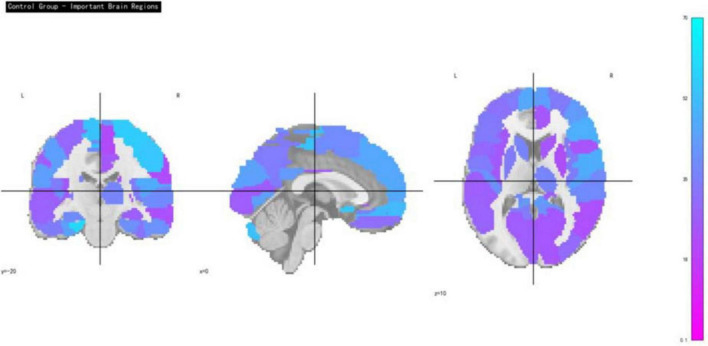
Control group key brain regions activation map. This figure visualizes, through Grad-CAM technology, the most important brain regions relied upon by the GCN model when making classification decisions for healthy controls. Darker colors indicate greater contribution of the brain region to healthy control classification. To facilitate independent visualization of each group, different pseudo-color schemes were used for the PD and control groups; quantitative comparisons should refer to the importance scores reported in Table 7.

To deeply investigate the decision-making basis of the trained GCN model for PD classification, this study employed the Grad-CAM algorithm to visualize the regions of interest focused on by the model. According to the AAL atlas, the 116 ROIs were divided into six major cerebral lobes based on their anatomical locations: frontal lobe, parietal lobe, occipital lobe, temporal lobe, limbic lobe, and subcortical regions. On this basis, key brain region activation maps for the PD group and healthy control group were generated, with the results shown in Figures 8, 9. The lobe-level importance distribution for the PD group and healthy control group is presented in Table 7. The PD group exhibited higher total importance scores in the frontal lobe (6.4692) and parietal lobe (4.9814), significantly higher than those in the temporal lobe, occipital lobe, limbic lobe, and subcortical regions. At the brain region level, among the top 10 most important brain regions in the PD group, the frontal lobe accounted for five regions and the parietal lobe accounted for three regions, with the right paracentral lobule contributing the most, followed by the left cuneus and right orbital part of the middle frontal gyrus, which also exhibited high importance. Correspondingly, quantitative analysis of the healthy control group revealed a relative reliance on connectivity patterns in the frontal lobe (7.3047) and parietal lobe (4.6864), while activation in the temporal lobe, occipital lobe, limbic lobe, and subcortical regions was lower. At the brain region level, among the top 10 most important brain regions in the healthy control group, the frontal lobe accounted for four regions and the parietal lobe accounted for four regions, with the left angular gyrus contributing the most, followed by the right orbital part of the superior frontal gyrus and the left parahippocampal gyrus, which also exhibited high activation intensity. To validate the statistical significance of the brain regions identified by Grad-CAM, independent sample *t*-tests were performed on the importance scores of the frontal and parietal lobes between the PD and healthy control groups. The results showed no significant difference in the frontal lobe, but a significant difference was observed in the parietal lobe, indicating that parietal regions played a more critical role in distinguishing PD patients from healthy controls.

**TABLE 7:** Lobe-level importance distribution for PD and control groups.

Lobe	PD group	Control group
Frontal lobe	**6.4692**	**7.3047**
Parietal lobe	4.9814	4.6864
Temporal lobe	3.3625	2.4416
Occipital lobe	1.5382	1.4375
Limbic lobe	1.3639	0.9099
Subcortical	1.3612	0.8563

The above Grad-CAM visualization results not only enhance the transparency of the model’s decision-making but, more importantly, reveal that the neuroanatomical basis underlying the model’s decisions is highly consistent with existing clinical neuroscience knowledge of PD, thereby validating the rationality of the model’s classification results from a computational perspective (Wang et al., 2021). The quantitative ROI-level statistics provide stronger empirical support for the identified key brain regions. It should be noted that the Grad-CAM results reveal the key brain regions that the model relies upon for classification decisions, rather than directly proving a pathological causal relationship between these regions and PD. These findings can serve as computational evidence for generating hypotheses and provide references for further neuroscientific validation. To evaluate the stability of the Grad-CAM results, we analyzed the consistency of key brain region identification across folds of the five-fold cross-validation. The results showed that the parietal and frontal lobes were consistently identified as key brain regions across all folds. Retraining and validation on the two independent external datasets also demonstrated that the identified key brain regions were highly consistent with those from the primary cohort, with the frontal and parietal lobes remaining the most dominant regions.

## 4 Discussion

This study proposed a DFC analysis framework based on a multi-view GCN, which achieved effective PD identification by independently constructing inter-subject similarity networks for each time window. Experimental results on the PPMI primary cohort demonstrated that the proposed method outperforms traditional SFC and clustering-based methods in classification accuracy, while also exhibiting good generalization capability on two independent external datasets. Combined with Grad-CAM interpretability analysis, the brain regions relied upon by the model’s decisions were further revealed, providing a basis for understanding the brain network mechanisms associated with PD. Compared with our previous work based on static FC (Lu and Zhao, 2026), the core differences of the present study lie in replacing static FC with multi-time-window DFC to capture time-varying information, replacing single static graph with independently constructed similarity networks for each sliding window, replacing single-view GCN with a multi-view fusion architecture, and adding Grad-CAM interpretability analysis. Both studies used the same PPMI primary cohort, while the present study additionally introduced two independent external datasets. Both used the same preprocessing pipeline. The previous work constructed a single similarity network based on static FC, whereas the present study constructs similarity networks independently for each window. The previous work employed a single-view GCN, whereas the present study adopts a multi-view GCN fusion architecture. Both used accuracy, precision, and recall as evaluation metrics.

Compared with the SFC-based graph convolution method, the proposed multi-view DFC framework achieved significantly improved classification performance. This advantage is primarily attributable to the effective capture of time-varying characteristics of functional connectivity by DFC, as well as the full utilization of temporal dimension information by the multi-view architecture. Traditional SFC methods rely solely on temporally averaged FC matrices, ignoring the dynamic reorganization characteristics of brain networks along the temporal dimension. In this study, DFC matrices for multiple time windows were constructed using the sliding window technique, and inter-subject similarity networks were independently constructed for each time window to form multi-view inputs. Regarding sliding window parameter selection, this study systematically compared five configurations of window size and stride ratio, demonstrating that model performance was optimal when the window size and stride ratio were 10/1, with shorter windows better capturing rapid dynamic fluctuations in FC and providing richer time-varying information to the model. Node embeddings for each time window were extracted using shared-weight GCN layers, and information from all views was fused at the node level, enabling the model to learn FC fluctuation patterns across multiple time scales and complementary differences in FC between groups of PD patients. In terms of inter-subject similarity network construction, the Gaussian similarity function significantly outperformed cosine similarity and median similarity in classification accuracy, with K = 30 yielding the optimal model performance. For model optimization, this study introduced Laplacian regularization constraints to suppress noise interference by enforcing smoothness of the graph structure while preserving the discriminative nature of key cluster structures, with parameter sensitivity analysis indicating that λ = 0.10 achieved the best model generalization. The above parameter sensitivity analysis provides important references for future studies. To validate the effectiveness of the multi-view architecture, this study compared three input strategies, with results showing that the proposed method significantly outperformed the approaches using only DFC matrices or k-means clustering in classification accuracy, fully demonstrating that independently constructing inter-subject similarity networks for each time window can effectively capture complementary characteristics of FC between individuals across different time scales. For model generalization capability evaluation, this study employed strict stratified five-fold cross-validation and conducted cross-dataset validation on two independent external datasets. Experimental results showed that the proposed model maintained high classification accuracy on both external datasets, significantly outperforming benchmark models such as SVM, MLP, GAT, and GT, verifying its robustness across different data sources and acquisition protocols. Regarding model interpretability, this study introduced the Grad-CAM algorithm for the first time into a multi-view DFC-based GCN classification framework, achieving visual analysis of the model’s decision-making basis. Results showed that the PD group exhibited higher total importance scores in the frontal and parietal lobes, with brain regions such as the right paracentral lobule, left cuneus, and right orbital part of the middle frontal gyrus contributing most to PD classification, a finding highly consistent with existing clinical neuroscience research. The healthy control group, in contrast, relatively relied on connectivity patterns in brain regions such as the left angular gyrus, right orbital part of the superior frontal gyrus, and left parahippocampal gyrus. These results not only enhance the transparency of the model’s decision-making but also provide computational support for the study of neural mechanisms in PD.

Despite the positive results achieved in this study, several limitations remain. First, although the generalization capability of the model was validated on multiple independent datasets, the overall sample size was still relatively limited; future work could further validate the model stability on larger-scale multi-center datasets. Second, although the sliding window parameters employed in this study were determined through sensitivity analysis, the optimal parameters may vary with different data acquisition protocols and disease types; future research could explore adaptive window selection methods. Third, the current framework primarily focuses on binary classification between PD and healthy controls; subsequent work could be extended to disease severity staging, subtype identification, or other neurodegenerative diseases. Fourth, although Grad-CAM provides visual interpretation of model decisions, it remains a *post-hoc* explanation method; future research could explore inherently interpretable frameworks that incorporate interpretability into model design. It should be noted that although our model achieves an advantage in classification accuracy, its recall remains lower compared to some baseline models. In clinical screening for PD, high accuracy is valuable for reducing overall misdiagnosis rates, while improving recall will be a key focus of future optimization. In summary, the proposed DFC analysis framework based on multi-view GCN, which independently constructs inter-subject similarity networks for each time window and fuses multi-view information combined with interpretability analysis, provides new methodological support for brain network analysis in PD. This method not only achieves favorable classification performance but also reveals candidate brain regions associated with PD and their dynamic variation patterns, advancing the application of deep learning in clinical neuroimaging analysis.

## 5 Conclusion

This study proposed a DFC analysis framework based on a multi-view GCN, which integrates multi-time-window DFC information into a unified graph learning model by independently constructing inter-subject similarity networks for each sliding time window. Combined with Laplacian regularization and Grad-CAM interpretability analysis, this framework achieved effective PD identification and provided computational evidence for understanding the underlying brain network mechanisms. Experimental results on the PPMI primary cohort and two independent external datasets demonstrated that the proposed method outperforms traditional SFC and clustering-based DFC methods in classification accuracy., including classification accuracy, precision, and recall. Through Grad-CAM interpretability analysis, this study further identified the critical roles of frontal and parietal lobe-related brain regions in model decision-making, providing computational evidence for understanding the brain network mechanisms of PD. This framework provides new methodological support for DFC-based brain network analysis in PD and can be extended to brain network analysis studies of other neuropsychiatric disorders.

## Data Availability

Publicly available datasets were analyzed in this study. This data can be found at: http://fcon_1000.projects.nitrc.org/indi/retro/parkinsons.html.

## Footnotes

1
www.ppmi-info.org
